# Thickness of individual layers at the macula and associated factors: the Beijing Eye Study 2011

**DOI:** 10.1186/s12886-019-1296-6

**Published:** 2020-02-12

**Authors:** Qian Wang, Wen Bin Wei, Ya Xing Wang, Yan Ni Yan, Jing Yan Yang, Wen Jia Zhou, Szy Yann Chan, Liang Xu, Jost B. Jonas

**Affiliations:** 1 Beijing Tongren Eye Center, Beijing Tongren Hospital, Beijing Ophthalmology and Visual Science Key Lab, Beijing Key Laboratory of Intraocular Tumor Diagnosis and Treatment, Capital Medical University, 1 Dong Jiao Min Xiang, Dong Cheng District, Beijing, 100730 China; 2 Beijing Institute of Ophthalmology, Beijing Ophthalmology and Visual Science Key Lab, Beijing Tongren Eye Center, Beijing Tongren Hospital, Capital Medical University, 17 Hougou Lane, Chong Wen Men, Beijing, 100005 China; 3 Department of Ophthalmology, Medical Faculty Mannheim of the Ruprecht-Karis-University Heidelberg, Mannheim, Germany

**Keywords:** Optical coherence tomography, Retinal thickness, Macular thickness, Retinal photoreceptor layer, Retinal ganglion cell layer, Beijing eye study

## Abstract

**Background:**

Diagnosis and follow-up of retinal diseases may be improved if the thickness of the various retinal layers, in addition to the total retinal thickness, is taken into account. Here we measured the thickness of the macular retinal layers in a population-based study group to assess the normative values and their associations.

**Methods:**

Using spectral-domain optical coherence tomographic images (Spectralis®, wavelength: 870 nm; Heidelberg Engineering Co, Heidelberg, Germany), we measured the thickness of the macular retinal layers in participants of the population-based Beijing Eye Study without ocular diseases and without systematic diseases, such as arterial hypertension, hyperlipidemia, diabetes mellitus, cardiovascular diseases, previous myocardial infarction, cerebral trauma and stroke. Segmentation and measurement of the retinal layers was performed automatically in each of the horizontal scans.

**Results:**

The study included 384 subjects (mean age:60.0 ± 8.0 years). The mean thickness of the whole retina, outer plexiform layer, outer nuclear layer,retinal pigment epithelium, inner retinal layer and photoreceptor layer was 259.8 ± 18.9 μm, 19.4 ± 3.9 μm, 93.4 ± 9.6 μm, 17.6 ± 1.9 μm, 169.8 ± 18.6 μm, and 90.0 ± 4.2 μm, respectively. In multivariable analysis, the thickness of the foveola and of all retinal layers in the foveal, parafoveal and perifoveal region decreased with older age (all *P* < 0.05), except for the thickness of the parafoveal outer plexiform layer which increased with age. Men as compared to women had higher thickness measurements of the photoreceptor layer and outer nuclear layer in all areas, and of all layers between the retinal nerve fiber layer and inner nuclear layer in the parafoveal area (all *P* < 0.05). The associations between the macular retinal layers thickness and axial length were not consistent. The inner plexiform layer was thicker, and the ganglion cell layer and inner nuclear layer were thinner, in the temporal areas than in the nasal areas,

**Conclusions:**

The associations between decreasing thickness of most retinal layers with older age and the correlation of a higher thickness of some retinal layers with male gender may clinically be taken into account.

## Background

The clinical introduction of optical coherence tomography (OCT), allowing the intravital non-invasive visualization of the various retinal layers with a spatial resolution of approximately 5 to 10 μm has markedly improved the possibilities in the diagnosis of retinal and optic nerve diseases [1, 2]. Besides the total retinal thickness, the thickness of the inner retinal layers, namely the retinal nerve fiber layer and the retinal ganglion cell layer, has routinely been measured for the diagnosis and follow-up examination of optic nerve diseases, in particular of glaucomatous optic neuropathy [3–6]. In contrast, the thickness of the middle and deep retinal layers has usually not routinely been assessed [7–12]. After automated algorithms have recently been developed, the thickness of the middle and deep retinal layers has been determined in healthy eyes, eyes with glaucoma and in eyes after optic neuritis [5, 7, 11–13]. Limitations of these studies were their design as hospital-based investigations and the relatively small size of the study populations. We therefore conducted the present study to measure the various retinal layers in a population-based investigation of healthy individuals.

## Methods

The population-based, cross-sectional Beijing Eye Study 2011 was carried out in 5 communities in the urban district of Haidian in the North of Central Beijing and in 3 communities in the village area of Yufa of the Daxing District in the South of Beijing [14, 15]. The only eligibility criteria for inclusion into the study were an age of 50+ years and living in the study region [16]. The study population consisted of 3468 (78.8%) individuals out of 4403 eligible individuals. The mean age was 64.6±9.8 years (median, 64 years; range, 50-93 years). Among the study participants, 1633 individuals (47.1%; 943 [57.7%] women) were from the rural region. The Medical Ethics Committee of the Beijing Tongren Hospital approved the study protocol, and all participants gave informed written consent.

Criteria for inclusion into the present study were the absence of systematic diseases such as arterial hypertension, hyperlipidemia, diabetes mellitus, manifest cardiovascular diseases, previous myocardial infarction, cerebral trauma and stroke, and the absence of ocular diseases such as glaucoma, diabetic retinopathy, status after cataract surgery, ocular trauma, retinal vascular occlusions, age-related macular degeneration, pigment epithelium detachment, retinal detachment, polypoidal choroidal vasculopathy and central serous chorioretinopathy. Myopia and incipient cataract not affecting the quality of the OCT images were no reason for exclusion of the individual.

The study participants underwent a series of examinations starting with an interview with standardized questions on the family status, level of education, income, depression, known major systemic diseases and quality of vision. The examination also included blood tests to measure the fasting blood concentrations of lipids, glucose and glycosylated hemoglobin A1c. The blood pressure and heart rate were assessed with the participants sitting for at least 5 minutes. Arterial hypertension was defined by a systolic blood pressure ≥140 mm Hg, a diastolic blood pressure ≥90 mm Hg, or by previous history of hypertension or use of antihypertensive medication. Body height and weight and the circumference of the waist and hip were recorded.

The ophthalmic examination included measurement of best-correcting refractive error, pneumatonometry, slit-lamp examination of the anterior segment and biometry of the right eye using optical low-coherence reflectometry (Lenstar 900 Optical Biometer, Haag-Streit, 3098 Koeniz, Switzerland). A slit-lamp examination performed by an ophthalmologist assessed lid abnormalities, corneal disorders, and peripheral anterior chamber depth. After medical dilatation of the pupil, photographs of the cornea and lens (slit-lamp digital photography, camera type BG-4, Topcon Medical Systems, Inc, Tokyo, Japan), and of the macula and optic disc (fundus camera type CR6-45NM, Canon Inc, Tokyo, Japan) were taken.

Retinal imaging was performed with spectral-domain OCT (SD-OCT) (Spectralis®, wavelength: 870nm; Heidelberg Engineering Co, Heidelberg, Germany) measuring a macular volume scan (25×30° field, 25 B-scan lines). Each scan line was based on 100 averaged scans. Segmentation of the retinal layers was performed automatically in each of the horizontal scans (Segmentation Technology; Heidelberg Engineering, Inc., Heidelberg, Germany). We used the nine segmentation lines: 1= inner limiting membrane; 2= posterior border of the retinal nerve fiber layer (RNFL); 3= posterior border of the retinal ganglion cell layer; 4= posterior border of the inner plexiform layer; 5= posterior border of the inner nuclear layer; 6= posterior border of the outer plexiform layer; 7= outer limiting membrane; 8= retinal pigment epithelium; 9= Bruch’s membrane. Using these nine segmentation lines, we measured the thickness of the retinal layers located between neighboring segmentation lines. These layers were the retinal nerve fiber layer (RNFL), ganglion cell layer (GCL), inner plexiform layer (IPL), inner nuclear layer (INL), outer plexiform layer (OPL), outer nuclear layer (ONL), retinal pigment epithelium (RPE), the inner retinal layer (IRL) as region between the inner limiting membrane and the outer limiting membrane, and the photoreceptor layer as the region between the outer limiting membrane and Bruch´s membrane. The automatically drawn segmentation lines were checked, and if needed interactively corrected by a trained examiner (QW).

Using the Early Treatment of Diabetic Retinopathy Study (ETDRS) map, we measured the thickness of the retinal layers in 9 regions. The macular area was divided into three concentric rings measuring 1 mm, 3 mm and 6 mm in diameter and which were centered on the fovea. The two outer rings with a diameter of 3 mm and 6 mm, respectively, were further divided into 4 equal regions (superior, inferior, nasal and temporal). The innermost ring with a diameter of 1 mm included the fovea (central subfield), while the 3 mm inner ring included the parafovea and 6 mm outer ring included the perifovea. The subfoveal choroidal thickness was additionally measured using the enhanced depth imaging modality of the OCT device. Only the right eye of each study participant was assessed. To assess the reproducibility of the measurement, we randomly selected 30 eyes of 30 participants and each parameter was measured three times in a masked manner with intervals of 2 weeks.

Statistical analysis was performed using a commercially available statistical software package (SPSS for Windows, V. 25.0, IBM-SPSS, Chicago, IL). We first calculated the mean and standard deviation of the main outcome parameters. i.e. the thickness of the retinal layers in the fovea, parafovea and perifovea areas. Secondly, we compared the thickness of the 10 retinal layers in each sector using the student t-test for paired samples. Applying Bonferroni´s correction, we corrected the dependence of the calculated *P*-value on the number of performed statistical comparisons. We assessed associations between the main outcome parameters and the other ocular or systemic variables in a univariate analysis. Finally, we carried out a multivariable analysis, with the retinal thickness parameters as dependent variable and with all those parameters as independent variables, which were significantly (*P*<0.05) associated with the retinal thickness parameters in the univariate analysis. We then dropped step by step those variables from the list of independent parameters, which either showed a high collinearity or which were no longer significantly associated with the outcome parameters. We additionally included the parameters of age, gender and axial length into the list of independent variables into the multivariate analysis, independently whether or not they were associated with the outcome parameters in the univariate analysis. The reason was that age, gender and axial length were associated with the retinal thickness in some previous studies [17]. Since the inner retinal layers were mostly absent in the fovea, only the thickness of the outer retinal layers was analyzed in the central subfield. We presented the standardized regression coefficient beta and the 95% confidence intervals (CIs) of the non-standardized regression coefficient B. To assess the reproducibility of the measurement, the intra-class correlation coefficient (ICC) and coefficient of variation were calculated. All *P*-values were two-sided and were considered statistically significant when the values were <0.05.

## Results

**Fig. 1:**
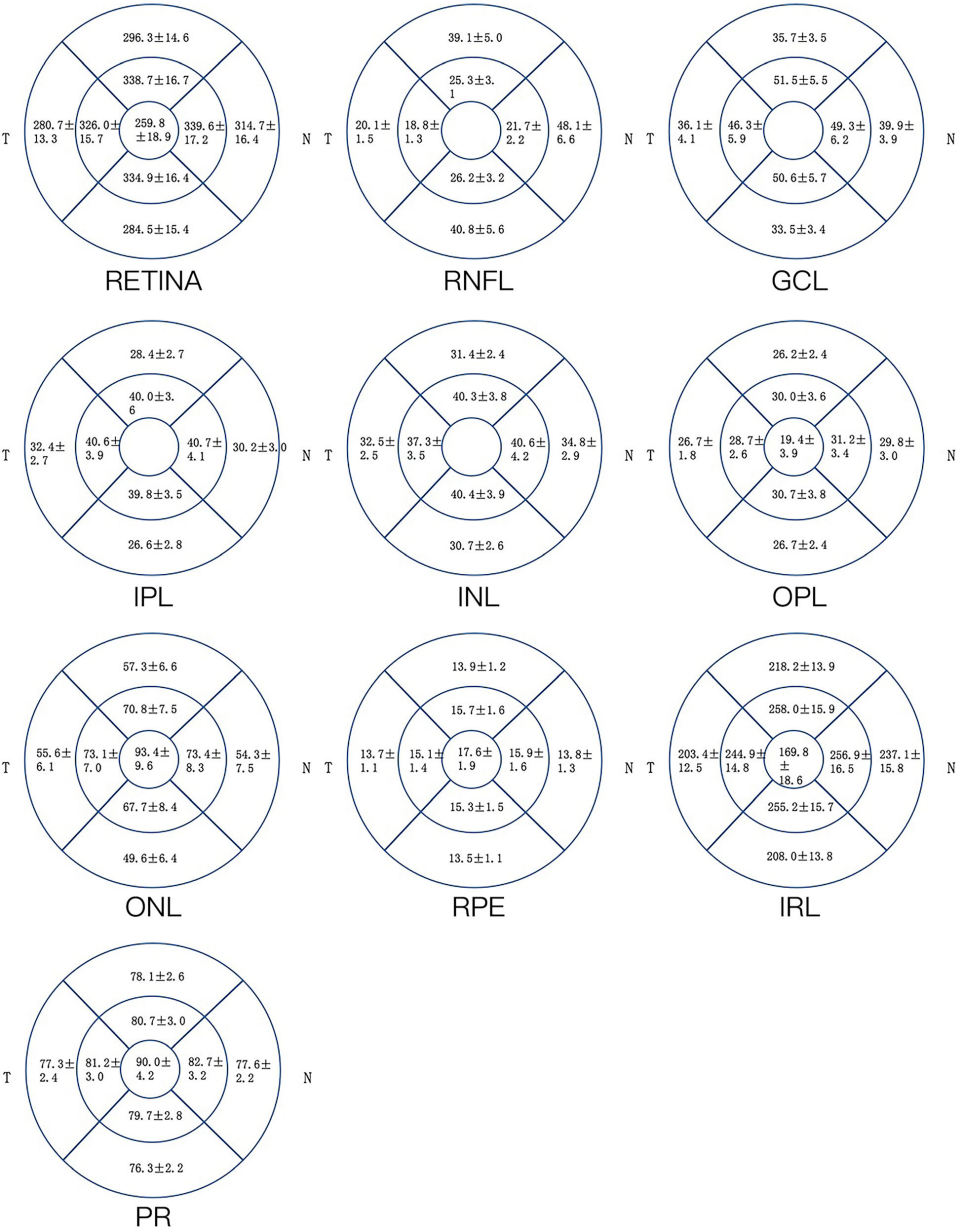
Mean (± standard deviation) thickness of the 10 retinal layers in the nine macular sectors. RNFL: retinal nerve fiber layer; GCL: ganglion cell layer; IPL: inner plexiform layer; INL: inner nuclear layer; OPL: outer plexiform layer; ONL: outer nuclear layer; RPE: retinal pigmented epithelium

Out of 3468 individuals participating in the Beijing Eye Study 2011, 3283 participants had undergone an examination by SD-OCT. Out of these 3283 individuals, 2867 participants were excluded since they did not fulfill the inclusion criteria due to systematic and/or ocular diseases including arterial hypertension and hyperlipidemia. Out of the remaining 416 participants with no systematic and/or ocular diseases, seven subjects were excluded due to poor quality of the OCT images and 25 subjects were excluded due to an inaccurate retinal layer segmentation that could not be corrected manually. Finally, 384 subjects (161 [41.9%] men) were included in this study. The mean age was 60.0 ± 8.0 years (median: 58.0 years, range: 50-84 years), the mean refractive error (spherical equivalent) was -0.13 ± 1.7 (median: 0.25 diopters, range: -11.38-5.50 diopters) and the mean axial length was 23.3 ± 1.1mm (median: 23.2mm; range: 20.2 - 28.9 mm) (Table 1). In 120 (31.3%) individuals, appropriate intra-retinal segmentation was achieved automatically, while the other remaining images needed additional manual corrections. The mean thickness values of the 10 retinal layers in the nine macular sectors are presented in Table 2 and Fig. 1.

**Table 1:** Measurements (Mean ± Standard Deviation) in participants included in the present study

Parameters	Mean ± Standard Deviation
n	384
Age (Years)	60.0 ± 8.0
Gender (men)	161 (41.9%)
mean refractive error (diopters)	−0.13 ± 1.71
Mean systolic blood pressure (mmHg)	122.0 ± 18.1
Mean diastolic blood pressure (mmHg)	67.7 ± 10.8
Mean fasting blood glucose (mmol/L)	5.3 ± 0.98
Mean heart rate (beats / minute)	73 ± 9
Mean waist circumference (mm)	85.6 ± 9.9
Mean hip circumference (mm)	98.4 ± 6.8
Mean body mass index (kg/m^2^)	24.5 ± 3.5
Mean high-density lipoprotein cholesterol concentration (mmol/L)	1.5 ± 0.4
Mean low-density lipoprotein cholesterol concentration (mmol/L)	3.4 ± 0.8
Mean triglyceride (mmol/L)	1.5 ± 1.1
Mean total cholesterol concentration (mmol/L)	5.1 ± 1.2
Mean creatinine (μmol/L)	65.2 ± 23.5
Mean C-reactive protein concentration (mg/L)	1.7 ± 3.2
Mean hemoglobin A1c (HbA1c %)	3.7 ± 0.7
Central cornea thickness (μm)	532 ± 31
Anterior chamber depth (mm)	2.5 ± 0.3
Lens thickness (mm)	4.5 ± 0.3
Axial length (mm)	23.3 ± 1.1
Subfoveal choroidal thickness (μm)	262 ± 94
Distance disc macula (mm)	4.9 ± 0.3

**Table 2:** Mean (± standard deviation) thickness of the 10 retinal layers in the foveal, parafoveal and perifoveal regions, and comparison with other studys

Study	Year	Sample	OCT instrument	Regions	Parameters									
					Retina (μm)	RNFL (μm)	GCL (μm)	IPL (μm)	INL (μm)	OPL (μm)	ONL (μm)	RPE (μm)	IRL (μm)	PRL (μm)
The Beijing Eye Study 2011	2018	384	Spectralis	Fovea	259.8 ± 18.9	–	–	–	–	19.4 ± 3.9	93.4 ± 9.6	17.6 ± 1.9	169.8 ± 18.6	90.0 ± 4.2
				Parafovea	334.8 ± 16.1	23.0 ± 2.0	49.4 ± 5.6	40.2 ± 3.5	39.6 ± 3.4	30.2 ± 2.2	71.3 ± 7.1	15.5 ± 1.4	253.7 ± 15.3	81.1 ± 2.8
				Perifovea	294.0 ± 14.0	37.0 ± 4.0	36.3 ± 3.4	29.4 ± 2.5	32.4 ± 2.3	27.4 ± 1.8	54.2 ± 6.2	13.7 ± 1.0	216.7 ± 13.2	77.3 ± 2.2
Appukuttan B [18]	2013	105	Spectralis	Fovea	260.1 ± 18.2									
				Parafovea	334.3 ± 16.9									
				Perifovea	293.2 ± 14.3									
Tewari HK [19]	2004	170	Stratus	fovea	181.2 ± 18.4									
Gella L [20]	2015	58	Copernicus	Central fovea	180.0 ± 18									65.5 ± 4.2
Demirkaya N [21]	2013	120	Topcon	Fovea		–	–	–	–	24.4 ± 5.1	–	18.3 ± 2.4	–	48.6 ± 3.9
				Parafovea		23.1 ± 1.8	50.6 ± 5.6	40.7 ± 3.3	39.6 ± 3.2	29.0 ± 3.5	–	18.2 ± 1.9	–	42.6 ± 3.6
				perifovea		35.4 ± 3.8	28.5 ± 3.0	37.0 ± 2.9	30.9 ± 2.5	25.2 ± 1.8	–	19.6 ± 1.8	–	38.4 ± 2.9
Loduca AL [11]	2010	15	Spectralis	Fovea		4 ± 3			23 ± 3	38 ± 7				

*RNFL* Retinal Nerve Fiber Layer; *GCL* Ganglion Cell Layer; *IPL* Inner Plexiform Layer; *INL* Inner Nuclear Layer; *OPL* Outer Plexiform Layer; *ONL* Outer Nuclear Layer; *RPE* Retinal Pigment Epithelium; *IRL* Inner Retinal Layer; *PRL* Photoreceptor Layer;

**Table 3:** Association (multivariable analysis) between the thickness of the various retinal layers and ocular and systemic parameters in the Beijing Eye Study; Beta: standardized regression coefficient; B: Non-standardized regression coefficient

Retinal Layer	Fovea			Parafovea			Perifovea		
	parameters	P-Value	Beta	Parameters	*P*-Value	Beta	Parameters	*P*-Value	Beta
Retina	Age	0.004	−0.14	Age	< 0.001	−0.34	Age	< 0.001	− 0.36
	Gender	< 0.001	− 0.36	Gender	< 0.001	− 0.25	Gender	0.001	− 0.18
				DDM	0.001	− 0.16	AL	< 0.001	− 0.30
RNFL	Gender	< 0.001	−0.20	Age	< 0.001	− 0.19	AL	< 0.001	0.25
	ACD	0.001	0.18	Gender	0.015	−0.13			
				AL	< 0.001	0.25			
GCL	Age	0.008	−0.15	Age	< 0.001	− 0.28	Age	< 0.001	− 0.37
	Gender	< 0.001	−0.21	Gender	0.042	−0.11			
	AL	0.006	0.16	AL	0.026	0.12	AL	< 0.001	−0.33
	SFCT	0.036	−0.12						
IPL	Age	0.024	−0.13	Age	< 0.001	−0.31	Age	< 0.001	−0.33
	Gender	< 0.001	−0.22						
	Pulse	0.025	−0.13	Gender	0.004	−0.14	AL	< 0.001	−0.32
	HDL	0.014	−0.15						
INL	Gender	< 0.001	−0.27	Age	0.017	−0.12	Age	< 0.001	−0.32
	AL	0.007	0.15	Gender	< 0.001	−0.23	AL	< 0.001	−0.28
	SFCT	0.005	−0.15						
OPL	Gender	0.018	−0.13	Age	0.004	0.15	No Associations		
	AL	< 0.001	0.21						
ONL	Gender	< 0.001	−0.20	Age	< 0.001	−0.19	Age	< 0.001	−0.28
	AL	0.047	−0.11	Gender	< 0.001	−0.24	Gender	< 0.001	−0.28
				AL	< 0.001	−0.32	AL	< 0.001	−0.37
RPE	Age	0.006	−0.15	SFCT	< 0.001	0.26	SFCT	< 0.001	0.35
	SFCT	0.001	0.18				Gender	0.041	−0.099
IRL	Gender	< 0.001	−0.33	Age	< 0.001	−0.31	Age	< 0.001	−0.30
				Gender	< 0.001	−0.23	Gender	0.007	−0.14
							AL	< 0.001	−0.30
PRL	Age	< 0.001	−0.25	Age	0.017	−0.13	Gender	< 0.001	−0.23
	Gender	0.003	−0.16	Gender	< 0.001	−0.21			
	AL	0.004	−0.16	AL	0.014	−0.13			

*RNFL* Retinal Nerve Fiber Layer; *GCL* Ganglion Cell Layer; *IPL* Inner Plexiform Layer; *INL* Inner Nuclear Layer; *OPL* Outer Plexiform Layer; *ONL* Outer Nuclear Layer; *RPE* Retinal Pigment Epithelium; *IRL* Inner Retinal Layer; *PRL* Photoreceptor Layer; *AL* Axial Length (mm); *SFCT* Subfoveal Choroidal Thickness (μm); *ACD* Anterior Chamber Depth (mm); *DDM* Distance disc macula; *HDL* High-Density Lipoproteins (mmol/L)

**Fig. 2:**
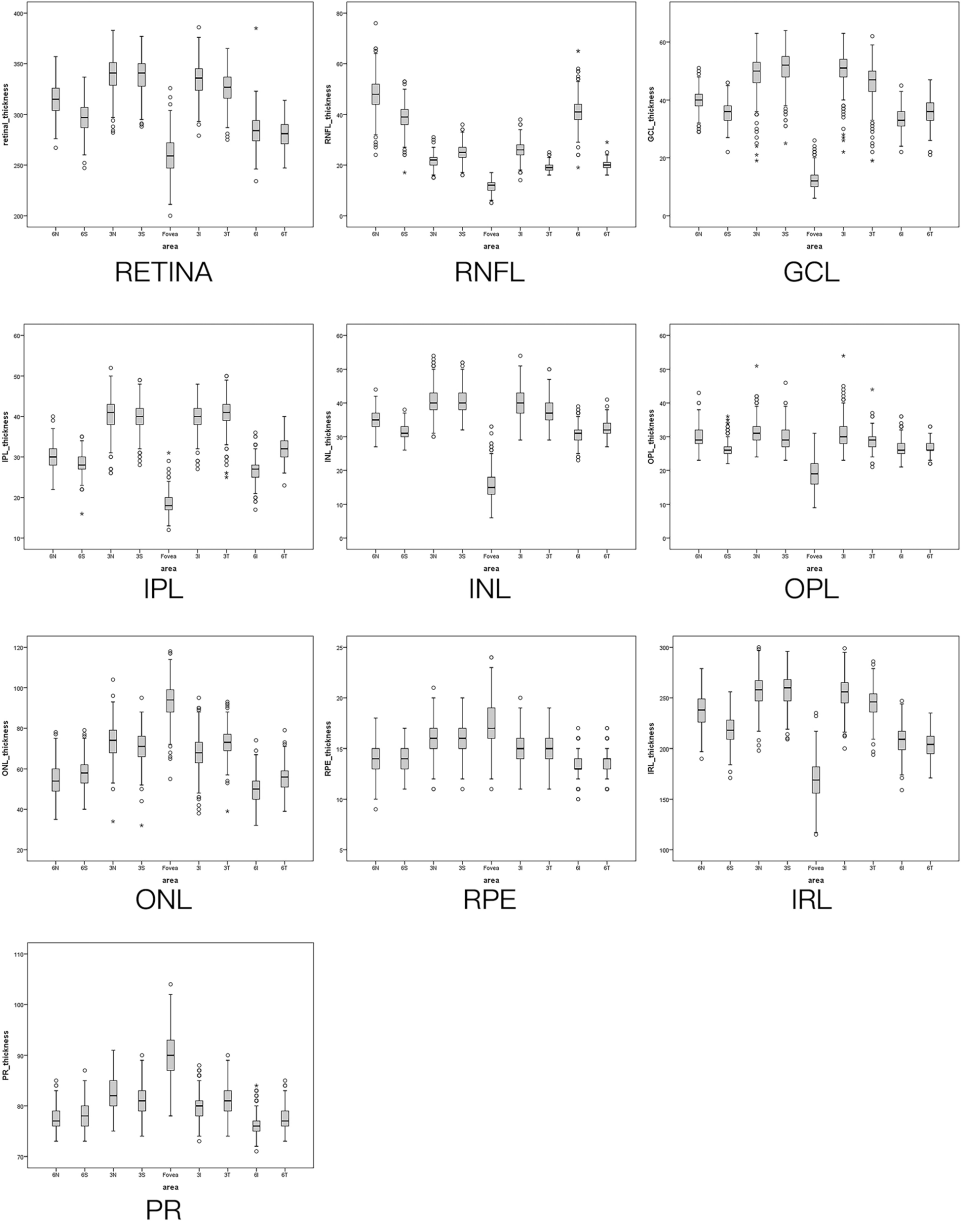
Thickness distribution of the 10 retinal layers in the nine retinal sectors

The retinal nerve fiber layer was thinnest in the temporal areas, and it was the thickest in the perifoveal area, nasal to the fovea and closest to the optic nerve head (all *P*<0.001). The retina as a whole (full-thickness) was thinnest in the central foveal area and it was thickest in the parafoveal regions. In the parafoveal and perifoveal areas, the total retinal thickness was thicker in the nasal areas than in the temporal areas (*P*<0.001). The ganglion cell layer, the inner plexiform layer and the inner nuclear layer were the thickest in the parafoveal regions. In the perifoveal retinal areas, the inner plexiform layer was thicker in the temporal areas than in the nasal areas. The ganglion cell layer and the inner nuclear layer were thicker in the nasal areas than in the temporal areas in both the parafoveal and the perifoveal retinal areas. The thickness of the outer nuclear layer, the retinal pigment epithelium layer and the photoreceptor layer was thickest in the central foveal sector and decreased with increasing distance to the foveal center (Fig. 2).

Men as compared to women had higher thickness measurements of the photoreceptor layer and ONL and of the inner retina in all areas, and of the RNFL, GCL, IPL and INL in the parafoveal area (all *P*<0.05 ) (Table 3). The association between the thickness of each retinal layer in the area of the fovea, the parafovea and the perifovea and the ocular and systematic parameters was listed in Table 3.

The intraclass correlation for the 10 thickness measurements of the various retinal layers in the nine macular sectors were all >0.90 with *P*-values <0.001, indicating a high intra-observer agreement.

## Discussion

The thickness measurements we obtained for the whole retina in different macular sectors were similar to those obtained in previous studies of healthy eyes, such as the investigations performed by Appukuttan and colleagues [18]. The measurements taken in our study were larger than those obtained by Tewari and associates and by Gella and colleagues [19, 20]. These discrepancies might have been due to differences in the OCT devices and in the segmentation methods applied. To cite an example, the definition of the outer retinal border differed in the four studies: it was the inner outer segment / RPE junction in Tewari’s study, the inner RPE surface in Gellai’s study, the outer RPE surface in Appukuttan’s study, and Bruch’s membrane in our investigation [18–20]. The regional distribution of the full retina thickness did not differ markedly among the studies, with the total retinal thickness being thicker in the nasal quadrant than in the temporal quadrant and being greater in the ETDRS inner ring than in the outer ring [17, 22–24].

The thickness of the RNFL, GCL, IPL and INL in each of the ETDRS sectors in our study were comparable to measurements taken in the studies carried out by Ooto and colleagues, while the INL thickness in Loduca’s study and in our study was similar and the thickness of the RNFL and OPL in our study were thinner than in Loduca’s study [11, 12]. Both studies revealed a similar regional distribution of the thickness of the various retinal layers study, except for that we did not observe the IPL being thicker in the nasal area than in the temporal area. Spraul and coworkers reported on a mean histological RPE thickness of 11.3 ± 1.4 μm in the foveal area and of 9.1 ± 2.0 μm in the perifoveal area [25]. These histomorphometric values were lower than those obtained in our study applying SD-OCT. The discrepancy between the measurements might have been mainly due to differences in the techniques employed, in addition to a tissue shrinkage caused by the histological tissue fixation. The mean thickness of the photoreceptor layer in the central foveal area as measured in our study population (90.0 ± 4.2 μm) was similar as determined in a previous investigation (88.8 ± 4.4 μm) by Alagöz and colleagues, while in both studies it was thicker than in another previous investigations (65.5 ± 4.2 μm) conducted by Gella and coworkers [20, 26]. The differences between the studies may be due to differences in the examination devices used and in the study populations.

The association between the thickness of the retinal layers and age has been addressed in previous studies. These studies demonstrated that the total retinal thickness and the thickness of the GCL, IPL and INL decreased with older age [12, 21, 24, 27, 28]. In a similar manner, our investigation reported an age-related reduction in the thickness of the whole retina, RPE and photoreceptor layer in the foveal region, of all retinal layers except for the OPL in the parafoveal area, and of the full retina, GCL, IPL, INL, ONL and IRL in the perifoveal region. In contrast to our and the other investigations, the UK Biobank Study revealed that, after exclusion of individuals with a history of ocular or systemic disease (diabetes or neurodegenerative diseases) and eyes with reduced vision, the mean central macular thickness in the central 1-mm ETDRS subfield (264.5 ± 22.9 μm) increased with older age and female gender, greater myopia, smoking, body mass index and white ethnicity [29]. As in our study, the macular thickness in other macular subfields decreased with older age and greater myopia. In another part of the UK Biobank study, Ko and colleagues reported that the mean retinal pigment epithelium-Bruch´s membrane thickness (26.3 ± 4.8 μm) in the central subfield showed an age-related thinning after an age of 45 years [30]. The findings obtained in our study suggest that the ageing process affected all retinal layers in all regions of the macula. These results were supported by the findings obtained in histological studies in which 0.3% to 0.6% of the retinal ganglion cells and retinal ganglion cell axons, 0.2% of the photoreceptor cones, 0.4% of the photoreceptor rods and 0.3% of the RPE cells were lost per year [31–33]. The thinning of the ONL with older age was first reported by Gartner and Henkind [34]. The histomorphometric result of an age-related decline being more pronounced for rods than for cones is in agreement with the finding obtained in the present study in which the ONL thickness in the parafoveal region and perifoveal areas decreased with older age, while the thickness of the ONL in fovea was not significantly correlated with older age [32]. In another histomorphometric study conducted by Curcio and colleagues, rod density decreased by 30% during a life span of 27 to 90 years, while changes in the cone density throughout this age span did not reveal consistent relationships to age [35].

In the present study, men as compared to women had higher thickness measurements of the photoreceptor layer and ONL and of the inner retina in all areas, and of the RNFL, GCL, IPL and INL in the parafovea area. These results were consistent with, and partially contradictory to, findings of previous studies. It has been reported that the full-thickness retina in the foveal region and in the parafoveal areas was thicker in men than in women [17, 36]. Ooto and colleagues reported that the thickness of the OPL and ONL combined was significantly thicker in men than in women [12].

**Fig. 3:**
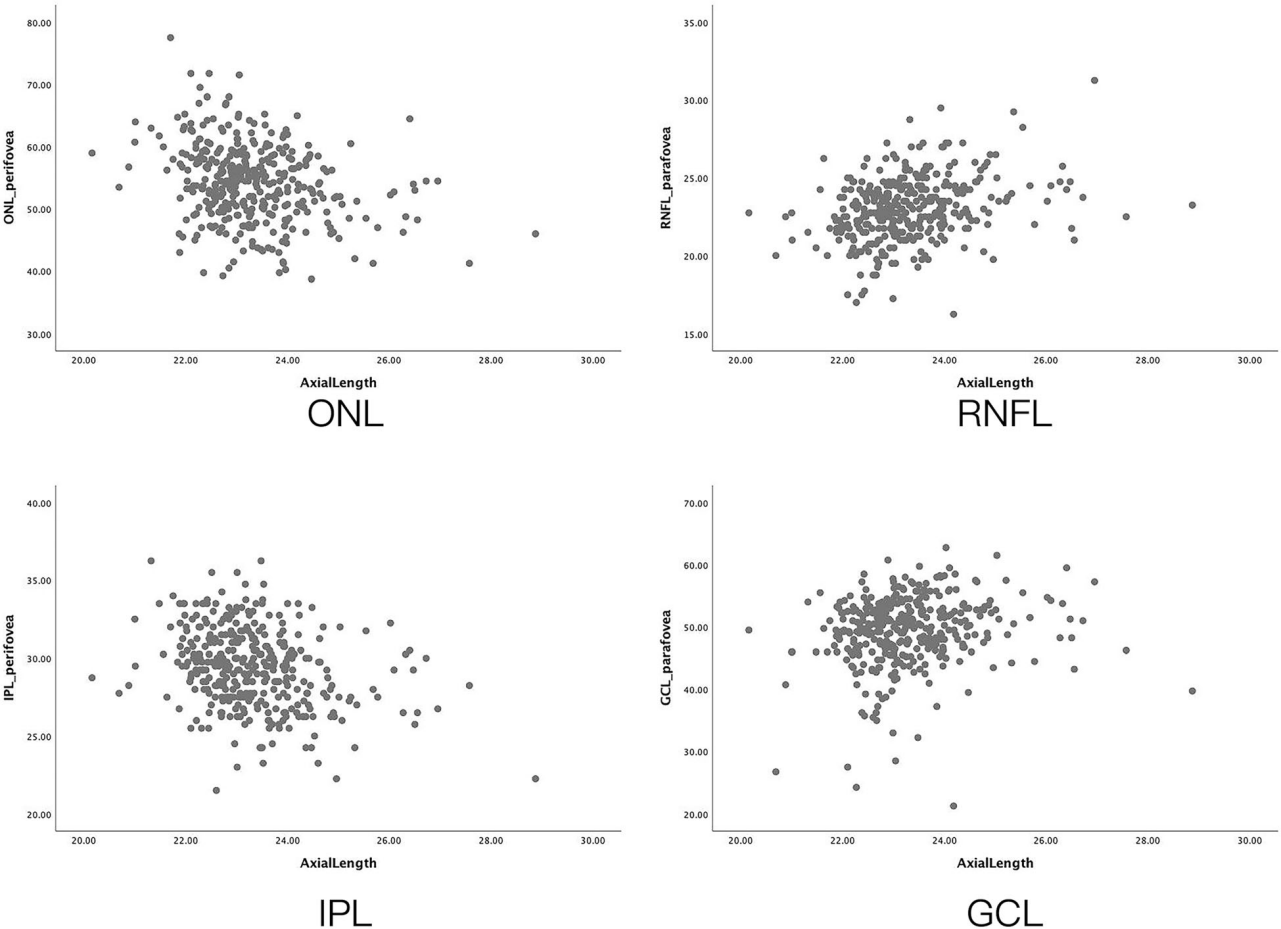
Relation between single retinal thickness and axial length

Associations between thickness measurements of the various layers and axial length were inconsistent. For most of the retinal layers, a thicker thickness was associated with a shorter axial length in the present study population, while in other layers, positive correlations were found (Fig 3). Previous studies mostly focused on the association between axial length and the full retinal thickness and had contradictory results when they included or excluded highly myopic eyes [36–38]. Our study included individuals with a refractive error which ranged from -11.38 diopters to 5.50 diopters (mean: -0.12 ± 1.7 diopters, median: 0.25 diopters).

In the present study, we did not find significant associations between the various retinal layer thickness measurements and systematic parameters, such as level of education, region of habitation, body height and weight, waist and hip circumstance, blood pressure, pulse, blood concentrations of high-density lipoprotein, low-density lipoprotein, cholesterol, triglycerides, glucose, glycosylated hemoglobin, and creatinine, aspirin intake, smoking package years, and alcohol consumption. This result might have been influenced by the pre-selection of the participants of the present study which included only normal participants.

Potential limitations of our study should be mentioned. First, as a population-based study, this study might have been vulnerable to a selection artifact. Second, our study included individuals with a range of refractive error between -11.38 diopters to +5.50 diopters (mean: -0.12 ± 1.7 diopters, median: 0.25 diopters). The results can therefore not be transferred on eyes with more extreme refractive errors. Future studies may be needed to investigate the relationship between the thicknesses of the various retinal layers and axial length, in particularly in eyes with extreme axial myopia. Finally, as mentioned above, the variety of OCT-devices has led to a marked variability in retinal thickness layer measurements, so that differences between studies might have been due to differences in the measurement devices.

## Conclusions

In conclusion, SD-OCT showed a relatively high agreement in the thickness measurements of the various retinal layers in healthy eyes. This study also demonstrated that retinal layer thickness measurements are associated with age, sex and axial length. These associations may be taken into account if, in addition to thickness measurements of the whole retina and of the retinal nerve fiber layer and retinal ganglion cell layer, thickness determinations of the middle and deep retinal layers are used for the diagnosis of retinal and optic nerve diseases.

## Data Availability

The datasets obtained and/or analyzed during the current study are available from the corresponding author on reasonable request.

## Abbreviations

CIs: Confidence intervals
ETDRS: The Early Treatment of Diabetic Retinopathy Study
GCL: Ganglion cell layer
ICC: Intra-class correlation coefficient
INL: Inner nuclear layer
IPL: Inner plexiform layer
IRL: Inner retinal layer
OCT: Optical coherence tomography
ONL: Outer nuclear layer
OPL: Outer plexiform layer
RNFL: Retinal nerve fiber layer
RPE: Retinal pigment epithelium
SD-OCT: Spectral-domain optical coherence tomography
